# Psychosocial Challenges and Opportunities for Youth With Chronic Health Conditions During the COVID-19 Pandemic

**DOI:** 10.2196/23057

**Published:** 2020-10-12

**Authors:** Anna Serlachius, Sherif M Badawy, Hiran Thabrew

**Affiliations:** 1 Department of Psychological Medicine Faculty of Medical and Health Sciences University of Auckland Auckland New Zealand; 2 Department of Pediatrics Feinberg School of Medicine Northwestern University Chicago, IL United States; 3 Division of Hematology, Oncology and Stem Cell Transplant Lurie Children’s Hospital of Chicago Chicago, IL United States

**Keywords:** COVID-19, coronavirus, pandemic, chronic illness, youth, adolescents, children, psychosocial, anxiety

## Abstract

School closures, altered access to health services, and economic stress during the COVID-19 pandemic have likely had an impact on the mental and physical well-being of youth worldwide, particularly among those with chronic health conditions (CHCs). A number of challenges and opportunities have emerged during the COVID-19 pandemic for youth with CHCs. Challenges include heightened anxiety, disrupted routines, academic and social stresses associated with school closure, increased risk of domestic violence and abuse, and reduced access to physical and psychosocial support. On the other hand, opportunities include reduced academic and social stress, increased time with families, reduced access to substances, easier access to health care using technology, and opportunities to build resilience. This viewpoint paper highlights both challenges and opportunities for youth with CHCs during the pandemic and offers recommendations for further research and clinical care.

## Introduction

The COVID-19 pandemic has disrupted the daily routines and peer interactions of millions of youth worldwide via mandated social distancing, rapid and sometimes repeated lockdowns, and prolonged school closures. Although little has been formally reported and the full impact of these measures may not be apparent for some time, it is likely that those with chronic health conditions (CHCs) who already face a disproportionate psychosocial burden will experience additional consequences [1]. Some of these consequences are likely similar to the general population and others will relate to their existing health issues. As not all effects of the pandemic are likely to be detrimental, we outline both the potential challenges and opportunities for youth with CHCs as well as recommendations for further research and clinical care.

## Psychosocial Challenges

Some of the key challenges likely to be faced by children and young people with CHCs during the COVID-19 pandemic include heightened anxiety regarding health and well-being; stress of disrupted routines; academic and social challenges associated with school closures; increased risk of family stress, domestic violence, and abuse; and reduced access to physical and psychosocial support. We discuss these challenges in this section in detail, and a summary of the challenges and strategies to address them is included in Textbox 1.

**Psychosocial challenges and strategies for children with chronic health conditions during COVID-19.**

**Heightened health anxiety**

Encourage developmentally appropriate communication with children about emotions and concerns

**Disrupted routines**

Establish predictable new routines

**School closures**

Encourage social/peer connection via technology

**Family stress/risk of domestic violence**

Prioritize monitoring and support of at-risk families and ensure the availability of safe houses for victims of domestic violence

**Reduced physical and psychosocial support**

Encourage families to use tele-health/digital therapies offered by health care providers

### Heightened Anxiety Regarding Health and Well-Being

For multiple reasons, including the demands of ill health, treatment, and readjustment to usual life following periods of medical treatment, children and young people with CHCs are at greater risk of developing psychological problems, especially anxiety [2]. Currently, services for these patients are limited, as are face-to-face and eHealth interventions, particularly those targeted toward health anxiety [3,4]. Partly due to the real dangers associated with the virus, the rapidity of lockdown, and the immediacy of social media, the COVID-19 pandemic has resulted in significantly increased rates of anxiety in the general population. Among those with life-threatening illnesses such as cancer, worries about social isolation, catastrophization about personal health, and guilt about family support have been even greater [5-7]. Studies of children from China have also identified clinginess, distraction, irritability, and fear of asking questions about the pandemic, more so in those who reside in highly affected regions [8]. It is not just those with physical health issues who are vulnerable. A recent UK survey of over 2000 young people with a history of mental health problems found that 51% of participants believed that their mental health had deteriorated due to the pandemic, and many reported increased psychological distress and loneliness [9]. Fortunately, there is emerging evidence suggesting that effective communication and distraction can help to protect children’s psychological health [8,10].

### Stress of Disrupted Routines

There is some evidence that children with attention-deficit/hyperactivity disorder (ADHD) and autism spectrum disorder (ASD), who usually thrive with predictability and routine, have been more affected by the disruption to routines during the pandemic than other groups. Children with ADHD have been found to display a greater level of symptoms that are related to family stress and can be reduced by establishing predictable new routines [11]. Those with ASD have also been found to exhibit greater behavioral concerns, especially in the face of pre-existing issues [12]. Increased physical activity has been suggested as one mechanism by which increased symptoms can be managed in these patients [13].

### Academic and Social Challenges Associated With School Closure

Children and young people spend a large proportion of their weekdays at school. Schools provide structure, intellectual stimulation, peer interaction, reliable meals, and access to recreational facilities and health care. Despite attempts to maximize online learning during lockdown, school closures are likely to have many unintended and potentially serious consequences on the psychological and physical health of children and young people [14,15]. Thousands, if not millions, of children from lower socioeconomic backgrounds may be disproportionately affected by school closures and experience food insecurity and inadequate or limited access to online learning [16,17]. Others are likely to engage in increased screen time and sedentary behaviors, just as during longer school holidays [18], placing themselves at risk of unhealthy weight gain [19]. Youth with pre-existing mental health conditions are especially reliant on psychological support services offered through schools and will be unable to access school counsellors, nurses, and social workers [20].

### Increased Risk of Family Stress, Domestic Violence, and Abuse

It is well established that increased family stress, financial insecurity, and cumulative risk exposure in childhood are associated with worse mental and behavioral outcomes in children [21,22]. Of particular concern, it is well documented that domestic violence is more likely in the face of chronic family stress such as caring for a child with a CHC. Moreover, chronic family stress further increases following crises such as natural disasters or disease outbreaks associated with economic stress [23-25]. Due to the focus on competing issues, vulnerable children including those with CHCs are often less likely to be identified via routine child health checks and by personnel such as health care professionals or school teachers. Coupled with the significantly reduced number of child protection assessments conducted during lockdown [26], it is likely that COVID-19–associated lockdown, school closures, and family financial insecurity will compound family stress levels and increase the incidence of domestic violence and child abuse [14,27], many of whom unfortunately may not be recognized to receive appropriate support.

### Reduced Access to Physical and Psychosocial Support

During the past few months, a significant decline in general practitioner appointments, specialized care, and pediatric emergency department attendance has been reported in many countries including the United Kingdom, Ireland, Germany, Canada, Australia, China, and Italy [28-35]. In some cases, delays in seeking treatment and lack of specialized care have had devastating effects on children with serious and life-threatening health complications including those of a psychological nature [36,37].

Other reported disruptions to health care services include disruptions to routine child health services, such as developmental screening, vaccinations, and well-child visits, that support psychosocial well-being [31,33]. There is also accumulating evidence to suggest that children with certain CHCs have been negatively impacted by changes to health care systems prioritizing the response to the pandemic, such as inflammatory bowel disease [38], pediatric cancer [39,40], and type 1 diabetes [41].

Previously limited psychological services for children and young people with CHCs are also likely to have been affected by COVID-19–related disruptions. Countries such as Singapore have reported diverting psychological resources for youth with eating disorders to only those considered most urgent and forging partnerships with community services to manage the decrease in access to psychological therapies [42], while other countries such as New Zealand have restricted face-to-face pediatric consult liaison services to inpatients at low risk of COVID-19. In the United Kingdom, 26% of young people with pre-existing mental health problems reported being unable to access psychological support services during the lockdown period [9].

## Psychosocial Opportunities

**Figure 1:**
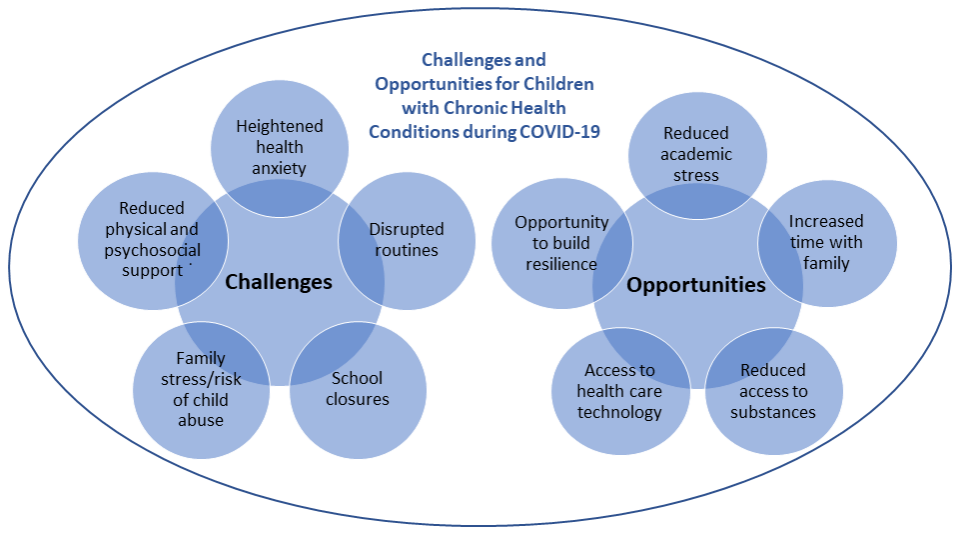
Opportunities and challenges for children with chronic health conditions during COVID-19.

Although children and young people with CHCs are at risk of all the previously mentioned challenges, they may also benefit in the following ways: reduced academic and social stress, increased time with families, reduced access to substances, easier access to health care using technology, and opportunities to build resilience. We discuss these opportunities in more detail in the following sections and a summary is included in Figure 1 along with related challenges.

### Reduced Academic and Social Stress

Although some degree of academic pressure may be essential for learning, the chronic stress of regular assignments, presentations, and examinations can have negative effects on students’ physical and psychological health, including precipitating conditions such as anxiety, depression, and eating disorders [43,44]. Associated with the developmentally congruent drive toward conformity, adolescent peer-related stress can also affect students’ health in a gender-related manner [45]. Youth with CHCs experience additional stresses related to disrupted education and peer relationships, readjustment during transitions in and out of the hospital, and the physical limitations of ill health [46]. During lockdown, all these issues are likely to occur less frequently. Additionally, for some, such as children with cystic fibrosis, the normalization of wearing masks may reduce the sense of difference and associated stigma [47].

### Increased Time With Families

Many children will be forced to spend weeks, if not months, with their families during lockdown. Given that domestic *social capital* has been shown to be more influential than school-related social capital and that it is associated with a reduced incidence of behavior problems [48], including in people with chronic illness [49], it is possible that many children and young people with CHCs will benefit from their parents being more available, being more involved in health care routines, and supporting them to deal with COVID-19–related or other health concerns. Family-based therapies for eating disorders [50] and other conditions may also be more effective in the context of greater parental availability. Additionally, evidence from Austria indicates that social connectedness can increase during lockdown and that it is associated with reduced distress and fatigue [51].

### Reduced Access to Substances

Up to 40% of young people with CHCs have issues related to substance abuse [52]. Pandemic-related anxiety, fear, and boredom are likely to increase the drive toward substance-related coping in this subgroup. Smoking and inhaling substances are particularly likely to increase the risk of contracting COVID-19 [53]. Fortunately, these risks are likely to be offset by reduced access to substances and, to a lesser extent, by lower financial independence and greater parental connection [54].

### Easier Access to Health Care Using Technology

Due to disruptions to routine services, health professionals worldwide have had to rapidly adopt or expand digital health care via Zoom (Zoom Video Communications, Inc), Skype (Skype Technologies), and other servers or platforms. Regulatory barriers to telemedicine have also been amended due to the urgency caused by the pandemic [55]. Despite some of the limitations associated with engagement and physical examination, the necessity for patients to have access to digital devices, and the potential loss of privacy for young people, digital health care is likely to have equitably increased access to health care for many families, especially those living rurally and with limited financial means. It is also likely to have reduced the anxiety experienced by some children in medical settings and allowed health professionals to gain a better understanding of their patients’ living circumstances. The pandemic may provide an additional opportunity to expand the use of existing eHealth interventions such as evidence-based internet cognitive behavioral therapy (iCBT)–based applications, medical support, and self-management interventions [56], and to identify ways for future interventions to improve outcomes for youth with CHCs.

### Opportunities to Build Resilience

Resilience has been defined as the ability of an individual to withstand adversity [57]. Despite the medically and socially related stresses they experience on a day-to-day basis, most children and adolescents with CHCs manage to live productive and effective lives, thereby demonstrating their inherent resilience [52]. The current pandemic is likely to provide them with additional opportunities to withstand novel concerns about their health; alterations to health care and other routines; increased family stress; and disappointment about missing out on schooling, peer interactions, and leisure activities. With adequate family, social, and health professional support, previous studies have proved that children with CHCs can surmount significant periods of difficulty [58].

## Recommendations for Future Research and Clinical Care

We offer the following recommendations and considerations for future research:

- Longitudinal studies of physical and psychosocial well-being, including rates of common mental health problems such as anxiety, depression, self-harm, and substance use disorders as well as rates of hospitalization and suicide, should be conducted across all age groups. Although a number of these studies are already underway and listed on websites such as Covid Minds [59], we noted that few studies are specifically targeted toward youth with CHCs.
- Prospective or retrospective analyses of high-risk subgroups including those with ADHD and ASD should be undertaken.
- Examination of the short- and long-term effects of school closure on personal stress, social relationships, and health care should occur.
- Analysis of the impact of the naturalistic increase in time spent with families, especially in regard to conditions such as eating disorders where social capital is integral to treatment, should be considered.
- Investigation of rates of domestic violence and abuse, particularly in families already experiencing chronic health-related stress, is necessary.
- In-depth qualitative analysis should be conducted regarding the views of patients of all ages, families, and health professionals regarding the use of digital health care, with a view to informing future service design and workforce development.
- Evaluation of the effectiveness of existing digital interventions should be planned as well as the co-design and development of locally and culturally acceptable new interventions for addressing COVID-19–related issues.
- Specific examination of resilience should be carried out using validated outcome measures, not merely assuming its existence in the absence of pathology.
- Economic analyses should be conducted to ascertain the direct and indirect costs of pandemic-related disruption and inform planning for future events of a similar nature.

In the meantime, to optimize the clinical care of children and young people with CHCs, we provide some resources and recommend readers to:

- Support families to access generic advice on how best to care for children and young people during the pandemic from organizations such as the World Health Organization [60] and, where available, more specific health-related advice via sources such as the International Society for Pediatric and Adolescent Diabetes [61], immunology and cancer services [62-64], and the Cystic Fibrosis Trust [65]
- Encourage families to maintain essential health care routines and to present early via appropriate channels when they have concerns about their children’s well-being
- Opportunistically screen for psychological issues, especially anxiety and depression, during clinical contacts using paper-based instruments such as the Generalized Anxiety Disorder scale-7 items and the Patient Health Questionnaire-9 item or electronic methods such as YouthCHAT [66,67]
- Refer those with identified issues early to available psychological services, either face-to-face or via tele-health
- Recommend the use of evidence-based digital self-help, iCBT, and peer support interventions; for an up-to-date list of these, refer to websites such as One Mind PsyberGuide [68]
- Encourage parents and health professionals to engage in self-care to reduce the likelihood of burnout and to sustain effective support of children and young people with CHCs during and following the pandemic

## Conclusion

Although the full impact of the COVID-19 pandemic on children and young people with CHCs might not be understood for a long time to come, increased awareness of the likely challenges and opportunities faced by this group and an integrated approach to their care [1] are likely to optimize their psychosocial well-being. Leveraging digital health interventions is key to addressing some of these challenges and opportunities in this vulnerable population [69]. The current circumstance also offers a unique opportunity to examine and improve a range of aspects pertaining to their care, and we sincerely hope that it provides the silver lining to a long dark cloud.

## Abbreviations

ADHD: attention-deficit/hyperactivity disorder
ASD: autism spectrum disorder
CHC: chronic health condition
iCBT: internet cognitive behavioral therapy
